# Supplementary material for: Association between Body Mass Index with Sugar-Sweetened and Dairy Beverages Consumption in Children from the Mexico–USA Border
Source: Int J Environ Res Public Health. 2022 May 25;19(11):6403. doi: 10.3390/ijerph19116403 (PMC9180521; doi:10.3390/ijerph19116403)
Supplement: Supplementary file 1 [file ijerph-19-06403-s001.zip › Supplementary File S1.pdf]

Supplementary File S1. Beverage Intake Questionnaire

Beverage Questionnaire

Instructions:
In the past month, please indicate your response for each beverage type by marking an "X" in the bubble for "how often" and "how much each time"
1) Indicate how often you drank the following beverages, for example, you drank 5 glasses of water per week, therefore mark 4-6 times per week
2) Indicate the approximate amount of beverage you drank each time, for example, you drank 1 cup of water 2 times per day, therefore mark 1 cup under "how much each time"

Subject ID ____________________

Date ____________________

	HOW OFTEN (MARK ONE)							HOW MUCH EACH TIME (MARK ONE)				
**Type of Beverage**	**Never or less than 1 time per week (go to next beverage)**	**1 time per week**	**2-3 times per week**	**4-6 times per week**	**1 time per day**	**2+ times per day**	**3+ times per day**	**Less than 6 fl oz (3/4 cup)**	**8 fl oz (1 cup)**	**12 fl oz (1 1/2 cups)**	**16 fl oz (2 cups)**	**More than 20 fl oz (2 1/2 cups)**
Water	O	O	O	O	O	O	O	O	O	O	O	O
100% Fruit Juice	O	O	O	O	O	O	O	O	O	O	O	O
Sweetened Juice Beverage/Drink (fruit ades, lemonade, punch, Sunny Delight)	O	O	O	O	O	O	O	O	O	O	O	O
100% Vegetable Juice (V8, etc.)	O	O	O	O	O	O	O	O	O	O	O	O
Whole Milk	O	O	O	O	O	O	O	O	O	O	O	O
Reduced Fat Milk (2%)	O	O	O	O	O	O	O	O	O	O	O	O
Low Fat/Fat Free Milk (Skim, 1%, Buttermilk, Soymilk)	O	O	O	O	O	O	O	O	O	O	O	O
Soft Drinks, Regular	O	O	O	O	O	O	O	O	O	O	O	O
Diet Soft Drinks/Artificially Sweetened Drinks (Crystal Light)	O	O	O	O	O	O	O	O	O	O	O	O
Sweetened Tea	O	O	O	O	O	O	O	O	O	O	O	O
Coffee, with cream and/or sugar (includes non-dairy creamer)	O	O	O	O	O	O	O	O	O	O	O	O
Tea or Coffee, black, with/without artificial sweetener (no cream or sugar)	O	O	O	O	O	O	O	O	O	O	O	O
Non-alcoholic or Light Beer	O	O	O	O	O	O	O	O	O	O	O	O
Beer, Ales, Wine Coolers	O	O	O	O	O	O	O	O	O	O	O	O
Hard Liquor (shots, rum, tequila, etc.)	O	O	O	O	O	O	O	O	O	O	O	O
Mixed Alcoholic Drinks (daiquiris, margaritas, etc.)	O	O	O	O	O	O	O	O	O	O	O	O
Wine (red or white)	O	O	O	O	O	O	O	O	O	O	O	O
Meal Replacement Shakes/Protein Drinks (Slimfast, shakes, etc.)	O	O	O	O	O	O	O	O	O	O	O	O
Energy Drinks (Red Bull, Rockstar, Full Throttle, etc.)	O	O	O	O	O	O	O	O	O	O	O	O
Other (list):	O	O	O	O	O	O	O	O	O	O	O	O
Other (list):	O	O	O	O	O	O	O	O	O	O	O	O

Virginia Polytechnic Institute and State University, 2008

Cuestionario de Consumo de Bebidas

Nombre: ____Fecha________________

Fecha de Nacimiento _____________Sexo___________ Grupo________

Instrucciones: Por favor marque con una X su respuesta en cada casilla sobre el tipo de bebida, la frecuencia de consumo y cantidad que consumió durante el mes pasado.

1. Indique la frecuencia con la que bebió las siguientes bebidas, por ejemplo, si bebió 5 vasos de agua por semana, debe marcar 4-6 veces por semana.
2. Indique la cantidad promedio aproximada de bebidas consumidas, por ejemplo, si consumió 1 taza de agua por día, marque la celda 1 taza por día.

	**Frecuencia de consumo (Marque con una X solo una para cada bebida)**							**Cantidad consumida en cada ocasión (Marque con una X solo una para cada bebida)**				
**Tipo de bebida**	Nunca	1 vez por semana	2-3 veces por semana	4-6 veces por semana	1 vez por día	2 veces por día	3 o más veces por día	Menos de 180 ml (3/4 de una taza)	240 ml (una taza)	360 ml (1 y ½ tazas)	480 ml (2 tazas)	Más de 600 ml (más de 2 ½ tazas)
Agua												
Jugo de fruta												
Jugos de verduras 100% (V8 etc.)												
Leche entera												
Leche baja en grasa												
Sodas o refrescos regulares												
Sodas o refrescos de dieta												
Té azucarado												
Café con crema y/o azúcar, (incluye crema no láctea)												
Te o café negro, con o sin edulcorante artificial (sin crema o azúcar)												
Cerveza sin alcohol o Light												
Cerveza, wine coolers												
Licores (whisky, ron, tequila, brandy, vodka, etc.)												
Mezcla de bebidas alcohólicas (Margaritas, Daiquiris, etc.,)												
Vino (rojo o blanco)												
Batido sustituto de una comida / Bebidas proteicas (slimfast, batidos, etc.)												
Bebidas energéticas (Red bull, Rockstar, Full throttel, etc.)												
Otras (Anotar):												
Otras (Anotar):												
