# Supplementary material for: Association between Body Mass Index with Sugar-Sweetened and Dairy Beverages Consumption in Children from the Mexico–USA Border
Source: Int J Environ Res Public Health. 2022 May 25;19(11):6403. doi: 10.3390/ijerph19116403 (PMC9180521; doi:10.3390/ijerph19116403)
Supplement: Supplementary file 1 [file ijerph-19-06403-s001.zip › Supplementary File S2.pdf]

Supplementary File S2. Statistical Analysis

Table S1

Descriptive Statistics

Age	Gender		N Statistic	Mean Statistic	Mean Std. Error
9	Girls	Weight	50	34.358	1.3742
		Height	50	1.343	.0099
		zScore	50	.5820	.15277
		Valid N (listwise)	50		
	Boys	Weight	33	39.676	2.3483
		Height	33	1.362	.0117
		zScore	33	1.2333	.16541
		Valid N (listwise)	33		
10	Girls	Weight	186	39.657	.6712
		Height	186	1.405	.0047
		zScore	186	.7274	.07935
		Valid N (listwise)	186		
	Boys	Weight	176	41.225	.8532
		Height	176	1.404	.0050
		zScore	176	.9393	.08850
		Valid N (listwise)	176		
11	Girls	Weight	118	44.315	.9240
		Height	118	1.473	.0072
		zScore	118	.6393	.09025
		Valid N (listwise)	118		
	Boys	Weight	117	42.921	.9070
		Height	117	1.465	.0072
		zScore	117	.6351	.10096
		Valid N (listwise)	117		
12	Girls	Weight	16	58.400	4.7754
		Height	16	1.521	.0167
		zScore	16	1.3225	.20615
		Valid N (listwise)	16		
	Boys	Weight	26	45.131	2.4325
		Height	26	1.509	.0185
		zScore	26	.1723	.27702
		Valid N (listwise)	26		

Descriptive Statistics

Age	Gender	NBM_EBM		N	Sum
9	Girls	EBM	NBM_EBM	21	21
			Valid N (listwise)	21	
		NBM	NBM_EBM	29	58
			Valid N (listwise)	29	
	Boys	EBM	NBM_EBM	20	20
			Valid N (listwise)	20	
		NBM	NBM_EBM	13	26
			Valid N (listwise)	13	
10	Girls	EBM	NBM_EBM	92	92
			Valid N (listwise)	92	
		NBM	NBM_EBM	94	188
			Valid N (listwise)	94	
	Boys	EBM	NBM_EBM	100	100
			Valid N (listwise)	100	
		NBM	NBM_EBM	76	152
			Valid N (listwise)	76	
11	Girls	EBM	NBM_EBM	52	52
			Valid N (listwise)	52	
		NBM	NBM_EBM	66	132
			Valid N (listwise)	66	
	Boys	EBM	NBM_EBM	52	52
			Valid N (listwise)	52	
		NBM	NBM_EBM	65	130
			Valid N (listwise)	65	
12	Girls	EBM	NBM_EBM	12	12
			Valid N (listwise)	12	
		NBM	NBM_EBM	4	8
			Valid N (listwise)	4	
	Boys	EBM	NBM_EBM	10	10
			Valid N (listwise)	10	
		NBM	NBM_EBM	16	32
			Valid N (listwise)	16	

Table S2

Descriptive Statistics

Gender	Age		N	Mean	
			Statistic	Statistic	Std. Error
Girls	9	Sugar_intake	50	38.8427	7.70289
		Energy	50	318.2976	62.61826
		SW_B_Kcal	50	174.2754	39.62097
		DB_Kcal	50	129.7950	29.09083
		SF_B	50	728.5200	107.57169
		SW_B_mL	50	515.2560	100.21808
		DB_mL	50	225.4320	50.44319
		Valid N (listwise)	50		
	10	Sugar_intake	184	42.4633	4.16699
		Energy	184	368.0491	32.59912
		SW_B_Kcal	184	181.9462	18.17906
		DB_Kcal	184	173.8221	19.02059
		SF_B	184	751.2587	47.78461
		SW_B_mL	184	516.7435	46.88575
		DB_mL	184	295.6174	33.06489
		Valid N (listwise)	184		
	11	Sugar_intake	116	49.7579	5.80147
		Energy	116	410.7847	43.81241
		SW_B_Kcal	116	213.2045	24.71074
		DB_Kcal	116	185.0820	25.12640
		SF_B	116	855.1966	60.26130
		SW_B_mL	116	586.9966	67.20302
		DB_mL	116	304.2000	40.25614
		Valid N (listwise)	116		
	12	Sugar_intake	16	39.3026	10.50938
		Energy	16	290.9901	67.77902
		SW_B_Kcal	16	191.7330	62.02022
		DB_Kcal	16	89.3691	11.67321
		SF_B	16	640.7250	143.51951
		SW_B_mL	16	573.8250	185.79260
		DB_mL	16	146.8500	18.71381
		Valid N (listwise)	16		
Boys	9	Sugar_intake	33	45.3623	8.19471
		Energy	33	281.3417	44.11350
		SW_B_Kcal	33	189.0753	34.85209
		DB_Kcal	33	77.1275	13.51280
		SF_B	33	593.7818	99.56640
		SW_B_mL	33	534.1091	88.88309
		DB_mL	33	129.6364	21.83518
		Valid N (listwise)	33		
	10	Sugar_intake	174	52.2634	3.55459
		Energy	174	380.9978	23.61043
		SW_B_Kcal	174	221.5141	15.92398
		DB_Kcal	174	142.3626	13.86428
		SF_B	174	710.0966	52.34386
		SW_B_mL	174	654.6690	41.46318
		DB_mL	174	240.3724	23.94965
		Valid N (listwise)	174		
	11	Sugar_intake	117	57.9373	6.90800
		Energy	117	442.3078	38.83258
		SW_B_Kcal	117	246.6993	30.42881
		DB_Kcal	117	177.7886	21.20380
		SF_B	117	836.4205	58.99788
		SW_B_mL	117	696.1744	72.59234
		DB_mL	117	296.6154	35.56739
		Valid N (listwise)	117		
	12	Sugar_intake	24	57.5740	12.03228
		Energy	24	396.1520	60.72683
		SW_B_Kcal	24	253.9444	54.84392
		DB_Kcal	26	169.8458	30.33641
		SF_B	26	808.2462	141.81332
		SW_B_mL	24	606.9833	107.09367
		DB_mL	26	279.9692	48.34926
		Valid N (listwise)	24		

Descriptive Statistics

NBM_EBM	Gender		N	Mean	
			Statistic	Statistic	Std. Error
EBM	Girls	Sugar_intake	175	44.6350	4.37253
		Energy	175	380.2962	33.18533
		SW_B_Kcal	175	190.6360	19.24642
		DB_Kcal	175	177.1298	20.35764
		SF_B	175	798.0206	49.89427
		SW_B_mL	175	566.3520	51.38856
		DB_mL	175	298.7383	34.89406
		Valid N (listwise)	175		
	Boys	Sugar_intake	182	52.9754	4.42597
		Energy	182	371.1680	24.68885
		SW_B_Kcal	182	228.9379	19.85732
		DB_Kcal	182	128.7489	11.94662
		SF_B	182	633.8044	42.85952
		SW_B_mL	182	638.0088	49.28883
		DB_mL	182	213.2901	19.49494
		Valid N (listwise)	182		
NBM	Girls	Sugar_intake	191	43.6912	4.16402
		Energy	191	363.3033	32.76774
		SW_B_Kcal	191	191.7803	18.81694
		DB_Kcal	191	159.0299	16.82467
		SF_B	191	756.3267	48.08438
		SW_B_mL	191	518.3497	48.94596
		DB_mL	191	267.1351	27.83945
		Valid N (listwise)	191		
	Boys	Sugar_intake	166	54.8777	4.43325
		Energy	166	417.3674	28.20777
		SW_B_Kcal	166	229.3659	19.36093
		DB_Kcal	168	173.2217	16.98177
		SF_B	168	873.0643	56.57124
		SW_B_mL	166	671.3277	46.23648
		DB_mL	168	293.2571	29.33940
		Valid N (listwise)	166		

Independent Samples Test

		Levene's Test for Equality of Variances		t-test for Equality of Means						
									95% Confidence Interval of the Difference	
		F	Sig.	t	df	Sig. (2-tailed)	Mean Difference	Std. Error Difference	Lower	Upper
Sugar_intake	Equal variances assumed	.023	.880	-2.243	712	.025	-9.74036	4.34280	-18.26660	-1.21413
	Equal variances not assumed			-2.242	709.070	.025	-9.74036	4.34432	-18.26962	-1.21110
Energy	Equal variances assumed	4.601	.032	-.725	712	.469	-21.77730	30.03589	-80.74680	37.19220
	Equal variances not assumed			-.729	686.394	.466	-21.77730	29.85276	-80.39100	36.83640
SW_B_Kcal	Equal variances assumed	.050	.822	-1.963	712	.050	-37.90890	19.31531	-75.83068	.01288
	Equal variances not assumed			-1.962	709.664	.050	-37.90890	19.31866	-75.83747	.01967
DB_Kcal	Equal variances assumed	5.537	.019	1.049	714	.294	17.58837	16.76109	-15.31854	50.49529
	Equal variances not assumed			1.055	682.933	.292	17.58837	16.66511	-15.13263	50.30938
SF_B	Equal variances assumed	.466	.495	.556	714	.578	27.61315	49.67125	-69.90601	125.13231
	Equal variances not assumed			.556	712.024	.579	27.61315	49.68005	-69.92375	125.15006
SW_B_mL	Equal variances assumed	.317	.574	-2.293	712	.022	-112.60066	49.10341	-209.00546	-16.19586
	Equal variances not assumed			-2.297	711.749	.022	-112.60066	49.01771	-208.83725	-16.36407
DB_mL	Equal variances assumed	5.792	.016	1.079	714	.281	30.57162	28.32966	-25.04777	86.19101
	Equal variances not assumed			1.085	683.984	.278	30.57162	28.16980	-24.73804	85.88127

Table S3

Correlations

		zScore	Energy	Sugar_intake	SW_B_Kcal	DB_Kcal	SF_B
zScore	Pearson Correlation	1	-.030	-.028	-.032	-.032	-.070
	Sig. (2-tailed)		.431	.454	.397	.397	.062
	N	722	714	714	714	716	716
[illegible]	[illegible]	[illegible]	[illegible]	[illegible]	[illegible]	[illegible]	[illegible]

Correlations

Gender			zScore	Energy	Sugar_intake	SW_B_Kcal	DB_Kcal	SF_B
Girls	zScore	Pearson Correlation	1	.011	-.002	-.017	.028	.056
		Sig. (2-tailed)		.836	.974	.743	.593	.287
		N	370	366	366	366	366	366
Boys	zScore	Pearson Correlation	1	-.083	-.062	-.054	-.101	-.185**
		Sig. (2-tailed)		.122	.249	.318	.058	.000
		N	352	348	348	348	350	350

Gender	Age			zScore	Energy	Sugar_intake	SW_B_Kcal	DB_Kcal	SF_B
Girls	9	zScore	Pearson Correlation	1	-.227	-.260	-.238	-.136	-.148
			Sig. (2-tailed)		.113	.068	.096	.346	.306
			N	50	50	50	50	50	50
		[illegible]	[illegible]	[illegible]	[illegible]	[illegible]	[illegible]	[illegible]	[illegible]
	10	zScore	Pearson Correlation	1	.090	.041	.020	.112	.085
			Sig. (2-tailed)		.225	.581	.791	.130	.254
			N	186	184	184	184	184	184
		[illegible]	[illegible]	[illegible]	[illegible]	[illegible]	[illegible]	[illegible]	[illegible]
	11	zScore	Pearson Correlation	1	.014	.049	.053	-.024	.112
			Sig. (2-tailed)		.878	.601	.570	.800	.232
			N	118	116	116	116	116	116
	12	zScore	Pearson Correlation	1	-.238	-.077	-.221	-.417	.361
			Sig. (2-tailed)		.374	.778	.410	.108	.170
			N	16	16	16	16	16	16
Boys	9	zScore	Pearson Correlation	1	-.290	-.311	-.301	-.167	-.163
			Sig. (2-tailed)		.101	.078	.089	.352	.365
			N	33	33	33	33	33	33
	10	zScore	Pearson Correlation	1	-.143	-.058	-.043	-.203**	-.018
			Sig. (2-tailed)		.059	.444	.570	.007	.819
			N	176	174	174	174	174	174
	11	zScore	Pearson Correlation	1	.095	.033	.037	.131	-.301**
			Sig. (2-tailed)		.310	.720	.689	.159	.001
			N	117	117	117	117	117	117
	12	zScore	Pearson Correlation	1	-.262	-.258	-.239	-.325	-.581**
			Sig. (2-tailed)		.215	.223	.260	.105	.002
			N	26	24	24	24	26	26

Correlations

Gender	NBM_EBM			zScore	Sugar_intake	Energy	SW_B_Kcal	DB_Kcal	SF_B
Girls	EBM	zScore	Pearson Correlation	1	.106	.068	.093	.003	.155*
			Sig. (2-tailed)		.163	.374	.223	.968	.041
			N	177	175	175	175	175	175
		Sugar_intake	Pearson Correlation	.106	1	.825**	.996**	.384**	.300**
			Sig. (2-tailed)	.163		.000	.000	.000	.000
			N	175	175	175	175	175	175
		Energy	Pearson Correlation	.068	.825**	1	.822**	.838**	.372**
			Sig. (2-tailed)	.374	.000		.000	.000	.000
			N	175	175	175	175	175	175
		SW_B_Kcal	Pearson Correlation	.093	.996**	.822**	1	.383**	.297**
			Sig. (2-tailed)	.223	.000	.000		.000	.000
			N	175	175	175	175	175	175
		DB_Kcal	Pearson Correlation	.003	.384**	.838**	.383**	1	.315**
			Sig. (2-tailed)	.968	.000	.000	.000		.000
			N	175	175	175	175	175	175
		SF_B	Pearson Correlation	.155*	.300**	.372**	.297**	.315**	1
			Sig. (2-tailed)	.041	.000	.000	.000	.000	
			N	175	175	175	175	175	175
	NBM	zScore	Pearson Correlation	1	-.080	-.049	-.091	-.009	.005
			Sig. (2-tailed)		.268	.502	.209	.901	.951
			N	193	191	191	191	191	191
		Sugar_intake	Pearson Correlation	-.080	1	.902**	.984**	.577**	.516**
			Sig. (2-tailed)	.268		.000	.000	.000	.000
			N	191	191	191	191	191	191
		Energy	Pearson Correlation	-.049	.902**	1	.887**	.866**	.519**
			Sig. (2-tailed)	.502	.000		.000	.000	.000
			N	191	191	191	191	191	191
		SW_B_Kcal	Pearson Correlation	-.091	.984**	.887**	1	.545**	.532**
			Sig. (2-tailed)	.209	.000	.000		.000	.000
			N	191	191	191	191	191	191
		DB_Kcal	Pearson Correlation	-.009	.577**	.866**	.545**	1	.394**
			Sig. (2-tailed)	.901	.000	.000	.000		.000
			N	191	191	191	191	191	191
		SF_B	Pearson Correlation	.005	.516**	.519**	.532**	.394**	1
			Sig. (2-tailed)	.951	.000	.000	.000	.000	
			N	191	191	191	191	191	191
Boys	EBM	zScore	Pearson Correlation	1	-.126	-.195**	-.121	-.212**	-.239**
			Sig. (2-tailed)		.090	.008	.103	.004	.001
			N	182	182	182	182	182	182
		Sugar_intake	Pearson Correlation	-.126	1	.876**	.995**	.143	.143
			Sig. (2-tailed)	.090		.000	.000	.054	.055
			N	182	182	182	182	182	182
		Energy	Pearson Correlation	-.195**	.876**	1	.874**	.597**	.121
			Sig. (2-tailed)	.008	.000		.000	.000	.104
			N	182	182	182	182	182	182
		SW_B_Kcal	Pearson Correlation	-.121	.995**	.874**	1	.135	.163*
			Sig. (2-tailed)	.103	.000	.000		.069	.028
			N	182	182	182	182	182	182
		DB_Kcal	Pearson Correlation	-.212**	.143	.597**	.135	1	-.024
			Sig. (2-tailed)	.004	.054	.000	.069		.750
			N	182	182	182	182	182	182
		SF_B	Pearson Correlation	-.239**	.143	.121	.163*	-.024	1
			Sig. (2-tailed)	.001	.055	.104	.028	.750	
			N	182	182	182	182	182	182
	NBM	zScore	Pearson Correlation	1	-.072	.023	-.088	.072	.008
			Sig. (2-tailed)		.360	.773	.258	.352	.916
			N	170	166	166	166	168	168
		Sugar_intake	Pearson Correlation	-.072	1	.810**	.994**	.203**	.267**
			Sig. (2-tailed)	.360		.000	.000	.009	.001
			N	166	166	166	166	166	166
		Energy	Pearson Correlation	.023	.810**	1	.786**	.736**	.310**
			Sig. (2-tailed)	.773	.000		.000	.000	.000
			N	166	166	166	166	166	166
		SW_B_Kcal	Pearson Correlation	-.088	.994**	.786**	1	.170*	.263**
			Sig. (2-tailed)	.258	.000	.000		.028	.001
			N	166	166	166	166	166	166
		DB_Kcal	Pearson Correlation	.072	.203**	.736**	.170*	1	.252**
			Sig. (2-tailed)	.352	.009	.000	.028		.001
			N	168	166	166	166	168	168
		SF_B	Pearson Correlation	.008	.267**	.310**	.263**	.252**	1
			Sig. (2-tailed)	.916	.001	.000	.001	.001	
			N	168	166	166	166	168	168

*. Correlation is significant at the 0.05 level (2-tailed).

**. Correlation is significant at the 0.01 level (2-tailed).
