# Supplementary material for: Association between Body Mass Index with Sugar-Sweetened and Dairy Beverages Consumption in Children from the Mexico–USA Border
Source: Int J Environ Res Public Health. 2022 May 25;19(11):6403. doi: 10.3390/ijerph19116403 (PMC9180521; doi:10.3390/ijerph19116403)
Supplement: Supplementary file 1 [file ijerph-19-06403-s001.zip › Supplementary File S3.pdf]

Universidad Autónoma de Baja California

FACULTAD DE DEPORTES

UABC/2018.02-E01

Ensenada, Baja California 15 de febrero de 2018

INVESTIGADOR: DR. LUIS MARIO GÓMEZ MIRANDA

La Junta Académica 230 Ejercicio Físico y Salud de la Facultad de Deportes de la Universidad Autónoma de Baja California, en reunión celebrada el día 14 de febrero de 2018, revisó las recomendaciones atendidas en el protocolo de investigación y el consentimiento informado del estudio denominado: "**Hábitos alimentarios y su relación con la composición corporal en niños de primaria en México y Estados Unidos**".

La junta académica resolvió al cumplirse con los lineamientos de la Declaración de Helsinki y los reglamentos éticos en los que se rige la Universidad Autónoma de Baja California, se dictamina como:

PROTOCOLO ACEPTADO PARA INTERVENCIÓN

Se extiende la presente para los fines necesarios, quedando de usted para cualquier duda o aclaración solicitada.

ATENTAMENTE
"POR LA REALIZACIÓN PLENA DEL HOMBRE"

UNIVERSIDAD AUTONOMA DE BAJA CALIFORNIA
POR LA REALIZACION PLENA DEL HOMBRE
FACULTAD DE DEPORTES

DR. ALBERTO JIMÉNEZ MALDONADO
Responsable de Junta Académica de Ejercicio Físico y Salud
Universidad Autónoma de Baja California
